# Stable Isotope Composition of Fatty Acids in Organisms of Different Trophic Levels in the Yenisei River

**DOI:** 10.1371/journal.pone.0034059

**Published:** 2012-03-28

**Authors:** Michail I. Gladyshev, Nadezhda N. Sushchik, Galina S. Kalachova, Olesia N. Makhutova

**Affiliations:** 1 Institute of Biophysics of Siberian Branch of Russian Academy of Sciences, Akademgorodok, Krasnoyarsk, Russia; 2 Siberian Federal University, Krasnoyarsk, Russia; Argonne National Laboratory, United States of America

## Abstract

We studied four-link food chain, periphytic microalgae and water moss (producers), trichopteran larvae (consumers I), gammarids (omnivorous – consumers II) and Siberian grayling (consumers III) at a littoral site of the Yenisei River on the basis of three years monthly sampling. Analysis of bulk carbon stable isotopes and compound specific isotope analysis of fatty acids (FA) were done. As found, there was a gradual depletion in ^13^C contents of fatty acids, including essential FA upward the food chain. In all the trophic levels a parabolic dependence of δ^13^C values of fatty acids on their degree of unsaturation/chain length occurred, with 18:2n-6 and 18:3n-3 in its lowest point. The pattern in the δ^13^C differences between individual fatty acids was quite similar to that reported in literature for marine pelagic food webs. Hypotheses on isotope fractionation were suggested to explain the findings.

## Introduction

Compound specific isotope analysis (CSIA) is comparatively new research tool for disentangling of natural food webs [1]–[3]. CSIA is expected to avoid many limits encountered when using more common trophic markers such as bulk tissue stable isotope or fatty acid (FA) analyses. CSIA appeared to be more successful than the other trophic markers in three cases: 1) when studied organisms cannot be physically isolated from each other; 2) if we need to trace quantitatively minor but qualitatively important component; 3) when different food sources have similar bulk carbon isotope and FA signatures.

The first case may take place in field studies when phytoplankton stable isotope signatures are inevitably measured together with those of bacteria, heterotrophic protists and detritus of different origins [4], [5]. For instance, using CSIA Boschker et al. [5] found that in the Scheldt estuary in the upper (freshwater) part isotope signatures of bacterial fatty acids were close to isotope ratios of bulk particulate organic carbon (POC), while algal FA ratios were comparatively depleted. In contrast, in the lower (marine) end of the estuary isotope signatures of bacterial and algal FA and POC were similar. These differences in FA signatures indicated that in the upper part of estuary bacteria preferentially used non-algal allochthonous carbon of terrestrial origin, while in the marine part of the estuary the local production by phytoplankton may be an important source for bacterial growth [5].

The second case is an identification of minor but essential food sources. Bulk carbon stable isotope analysis provides information on food sources most significantly contributing to consumers' diet. However, a minor dietary source may play a significant role as a supplier of essential compounds. Thus, in identifying only the quantitatively important organic matter sources, bulk carbon stable isotope analysis may underestimate the functional importance of minor dietary sources [3]. For instance, Koussoroplis et al. [3] found that the estuarine fish *Liza saliens* during settlement switched from planktonic to benthic food sources and δ^13^C signatures of bulk carbon increased accordingly. However, fatty acid 22:6n-3, which is known to be especially essential for fish, kept depleted planktonic δ^13^C signatures. Consequently, although the production of settled fish was essentially supported by benthic dietary sources, as confirmed by bulk carbon stable isotope results, minor reliance on planktonic dietary sources was required to provide fish with 22:6n-3 [3].

The third case arises when food sources to be differentiated have similar bulk stable isotope signatures and similar FA composition. For instance, Budge et al. [2] using CSIA of fatty acids, especially 16:4n-1, differentiated sea ice diatoms from pelagic diatoms, which could not be discriminated by the other methods. Thereby it was found, that 24–71% of FA in higher trophic levels, up to seals and birds, in some arctic seas in April–May was derived from ice algae, rather than from pelagic phytoplankton [2].

It must be stressed that the cited field works were based on the essential premise that δ^13^C isotope ‘signal’ of individual FA is actually transmitted from the diet to the consumer [6]. However, experimental tests are still rare and did not necessarily support this essential assumption [6]–[8]. Moreover, FA δ^13^C values of zooplankton and fish were not directly related to those of particulate organic matter in a marine field study [4]. Hence, more studies are now required to understand which information FA-CSIA could provide for tracing food webs.

The isotopic composition of individual fatty acids is known to be controlled by the nature and availability of the carbon source and the isotopic fractionation accompanying metabolism and biosynthesis in the living organisms [6]–[9]. For instance, the kinetic isotope effect during the pyruvate dehydrogenase reaction accounts for the ^13^C depletion of the lipid fraction observed in organisms as they exist in nature [10]. Desaturation and chain elongation of fatty acids also may have an associated kinetic isotope effect, and this effect should have made long-chain highly unsaturated fatty acids (HUFA) lighter, not heavier, than their precursors [6], [9], [11]. Nevertheless, such HUFA as eicosapentaenoic fatty acid (20:5n-3, EPA), and docosahexaenoic fatty acid (22:6n-3, DHA), in pelagic food webs may have relatively high δ^13^C value compared to their precursors, C18 acids [4], [6].

In contrast, essential fatty acids (EFA), such as linoleic acid (18:2n-6, LA) and α-linolenic acid (18:3n-3, ALA), which are not synthesized *de novo* by consumers, are generally expected to have the same isotopic signatures in consumer tissues as those of the food source [4], [6]. However, even the essential fatty acids were found to be generally ^13^C-depleted compared with their counterpart in the corresponding diet in field and laboratory pelagic food webs [4], [6]. Thus, variations in isotope fractionation in some fatty acids are difficult to explain with current theories of biosynthesis and isotope fractionation, and more work is needed.

In field studies, if researchers when studying unknown food webs faced with depleted EFA in consumers compared to probable food source, they often explain it by an additional “unique” input to the animal tissue of these fatty acids [9], [12]. However, Veefkind [4] found out, that the depletion of essential FA in consumers compared to their food source appeared to be a wide-spread phenomenon in marine environment. He revealed a reproducible pattern in the δ^13^C differences between individual fatty acids, which was subsequently passed on from phytoplankton to higher organisms through feeding. This pattern, besides the above depletion, consisted in declining of δ^13^C of C_18_ fatty acids with an increasing number of double bonds and in relatively high δ^13^C values for the 20:5n-3 and 22:6n-3.

If a certain pattern of FA isotope signatures exists in aquatic ecosystems, it would be potentially important for better deciphering of field data of CSIA, because at present there are too many biases in interpretation of δ^13^C values of individual FAs [1], [12]. Thus, the aim of our work was to study if a pattern in the δ^13^C differences between individual fatty acids, which was subsequently passed on from primary producers to consumers, exists in a community of river benthos.

## Materials and Methods

### Sampling sites

The Yenisei River is the largest river in Russia, and the eighth largest in the world with respect to its average annual discharge ca. 600 km^3^. The main hydroecological features of the river are given elsewhere [13]. Briefly, the main hydrochemical peculiarities of the Yenisei are a low turbidity and 100% saturation level of dissolved oxygen.

Two sample sites were situated in the middle section of the river, 30 km downstream of the dam of Krasnoyarsk Hydroelectric Power Station (upstream Krasnoyarsk city), and 30 km downstream Krasnoyarsk city (52 km downstream from the first site), respectively. Both sites are little affected by human activity and have the same ecological features, both biotic and abiotic. The width of river at both the sampling sites is about 1 km. The surface of the river is ice-free throughout the winter because of the discharge of deep warm waters from the upstream reservoir. Water temperature ranged from 5–10°C in spring and summer and 0–5°C in autumn and winter. The river banks are covered with taiga, i.e., evergreen coniferous trees, which grow on high rocky banks and don't shadow the sites. Flying insects are practically absent on the surface of such large river even in summer, not to mention the other seasons. Thus, the river communities depend on autochthonous organic inputs only. Flow velocity at the sites is high, about 2 m s^−1^, and there are no sediments (detritus) on the pebbly bottom.

Bottom pebbles are covered with peryphyton (epilithic biofilms), primarily composed of microalgae. The epilithic microalgae at the site are described in details elsewhere [14]–[16]. Briefly, the microalgal biomass was very high in spring and early summer, reaching *ca.*1 000 g m^−2^ wet weight, at the expense of green algae of *Ulothrix* genus. Later mostly diatoms comprised phytobenthos, and several dominant species replaced each other over the year, i.e., genera *Gomphonema* and *Didymosphaenia*, and species *Rhoicosphaenia abbreviata* (Kutz.) Grun and *Cocconeis placentula* Ehr. In summer the phytobenthos biomass varied from about 5–50 g m^−2^. In the late autumn and winter, the phytoperiphyton biomass varied from about 0.1 to 1 g m^−2^, and cyanobacteria became the dominant species. The epilithic microalgae had a very high gross primary production, up to 95.1 g C m^−2^ day^−1^ [17]. Besides the periphytic microalgae, clumps of water moss, *Fontinalis antipyretica* L. ex Hedw., were characteristic of the sampling site.

The zoobenthos of the study site is described in detail elsewhere [14], [18], [19]. Briefly, their biomass reaches up to *ca.* 40 g m^−2^ wet weight, and *Eulimnogammarus* (*Philolimnogammarus*) *viridis* Dybowsky was by far the dominant species. The subdominant species were larvae of Trichoptera (*Apatania crymophila* McLachlan). Other taxa, chironomid and ephemeropteran larvae composed very small part of zoobenthos biomass.

We sampled phytoperiphyton (epilythic biofilms) and zoobenthos about monthly in October, 2008–January, 2011 in the littoral at about 0.5 m depth. Dominant fish species, Non-living fish, Siberian grayling, *Thymallus arcticus* Pallas, were obtained from local fishermen about bimonthly. Thus, no permission/experimentation care for the animals was needed.

### Fatty acid analysis

Detailed description of fatty acid analyses of the epilithic microalgae, the zoobenthos and the fish are given elsewhere [18], [20]. Briefly, lipids from epilithic biofilms, zoobenthos (after a day gut empting) and fish muscle tissue samples were extracted with chloroform/methanol (2∶1, v/v) three times simultaneously with mechanical homogenization with glass beads. Methyl esters of fatty acids (FAMEs) were prepared in a mixture of methanol–sulfuric acid (20∶1, v/v) at 85°C for 2 h. FAMEs were then analyzed using a gas chromatograph–mass spectrometer (model 6890/5975C, “Agilent Technologies”, USA) equipped with a HP-FFAP capillary column (30 m length, 0.25 mm internal diameter). Peaks of FAMEs were identified by their mass spectra, compared to those in the database (“Agilent Technologies”, USA) and to those of available authentic standards (Sigma, USA). To determine double bond positions in monoenoic and polyenoic acids, GC–MS of dimethyloxazoline derivatives of fatty acids was used [21]. Prior to the GS-MS analysis a part of the lipid extract of some samples of gammarids was separated on a TLC 6×6 cm microplate with solvent system: hexane∶diethyl ether∶acetic acid (85∶15∶1, v/v/v). Lipid spots were identified by comparison their *R_f_* with those of standards (Sigma-Aldrich, USA, Serva, Germany). Silica gel, containing triacylglycerols (TAG) was scraped from the plates and the lipids were redissolved for following FA analysis, described above. To answer the main question of our study we compared FAs of total lipids in all the groups of organisms. TAG FAs and FAs of total lipids of the gammarids were compared to see a degree of probable differences in the isotope composition between total FAs and FAs in certain classes of lipids.

### Stable isotope analyses

The stable isotope analysis (SIA) of bulk carbon and nitrogen was described in details elsewhere [22]. Briefly, samples were analyzed with a continuous flow isotope ratio mass spectrometer (CF-IRMS), model Delta V Plus (Thermo Scientific Corporation, USA) interfaced with a elemental analyzer (Flash EA 1112 Series, Thermo Electron Corporation, USA). Dry helium of 6.0 grade was used as carrier gas for sample introduction. Reference tanks for N and C isotopes were made of pure N_2_ (5.5 grade, 99.9995%) and CO_2_ (4.5 grade, 99.995%).

Stable isotope data were conventionally expressed in the per mil delta notations, δ^13^C for carbon relative to Vienna Pee Dee Belemnite, and δ^15^N for nitrogen relative to atmospheric N_2_ [23]. The accuracy and precision of the measurement was verified daily with the secondary reference material USGS40 from International Atomic Energy Agency (L-glutamic acid, δ^15^N = −4.5‰ and δ^13^C = −26.39‰). Analytical reproducibility was ±0.2‰ for C and ±0.3‰ for N. The laboratory standard (Urea, Thermo) was analyzed every 12 samples. Samples were analyzed in duplicate or triplicate when sufficient material was available.

Trophic position (TP) was calculated conventionally:
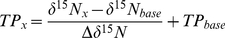
(1)where δ^15^N_x_ is the isotope ratio of the taxon in question, Δδ^15^N is the trophic enrichment (fractionation) constant, δ^15^N_base_ and TP_base_ are the average δ^15^N and trophic position of the baseline, respectively [24]. The constant Δδ^15^N = 3.4‰ and TP_base_ = 2 [24], [25]. The taxonomic group of zoobenthos with the lowest δ^15^N was selected as the baseline for estimating the TPs of other taxa [25].

### Compound specific isotope analysis

Generally, the conditions of gas chromatographic analysis of isotopic ratio of FAMEs were identical with standard GC-MS analysis. Carbon isotopic composition of individual FAME was determined with an isotope ratio gas-chromatograph -mass spectrometer (GC-IRMS) system: a Trace GC Ultra (Thermo Electron) gas-chromatograph was interfaced with a Delta V Plus IRMS (Thermo Fisher Scientific Corporation) via a type-III combustion interface and installed with a Thermo DB-FFAP column (50.0 m length, 0.25 mm ID, 0.25 µm film thickness). Conditions of the IRMS instrument were follows: electron ionisation, 100 eV, 3 Faraday cup collectors m/z 44, 45 and 46, CuO/NiO combustion interface maintained at 940°C. The samples were injected in a split mode (inlet temperature 250°C, carrier gas, helium, constant flow rate of 1 ml min^−1^, oven temperature raised from 160 to 230°C. The isotopic values of the peaks produced by combustion of the chromatographically separated compounds were calculated using CO_2_-spikes of known isotopic composition, introduced directly into the source three times at the beginning and end of every run. An alkane references mixture of known isotopic composition (C15, C20, C25, Chiron, Norway) was run after every three-four samples to check the accuracy of the isotopic ratios determined by the GC-IRMS.

Stable carbon isotope ratios for individual fatty acids were calculated from FAME data by correcting for the one carbon atom in the methyl group that was added during methanolis [9], [26]:
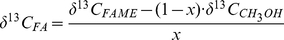
(2)where δ^13^C_FA_ is the isotopic composition of the free fatty acids, δ^13^C_FAME_ is the isotopic composition of the fatty acid methyl ester, *x* is the fractional carbon contribution of the free fatty acid to the ester and δ^13^C_CH3OH_ is the isotopic composition of the methanol derivatization reagent (−46.8‰ in our work). The isotopic composition of the used methanol was determined by the same GC-IRMS system working isothermally at 65°C.

### Statistics

Standard errors, Kolmogorov-Smirnov one-sample test for normality, Student's *t*-test, Wilcoxon matched pairs test, Fisher's LSD (least significant difference) *post-hoc* test and Pearson's product-moment correlation were calculated conventionally [27], using STATISTICA software, version 9 (StatSoft Inc., Tulsa, OK, USA).

## Results

Fatty acid composition of total lipids of the studied organisms (groups) is given in **Table 1**. Besides 16:1n-7, comparatively high levels of 18:2n-6, 18:3n-3, 18:4n-3 and 20:5n-3 were characteristic of the phytoperiphyton (**Table 1**). All the animals had high levels of 18:1n-9. The trichopterans had high levels of 18:2n-6, 18:3n-3 and18:4n-3, but very low level of 22:6n-3 compare to the other animals (**Table 1**). Very high level of 20:5n-3 was characteristic of the gammarids, and extremely high level of 22:6n-3 was characteristic of the grayling (**Table 1**). The water moss had comparatively high levels of 18:2n-6, 18:3n-3 and 20:4n-6, and also contained specific acetylenic fatty acids, 6a,9,12-18:3 and 8a,11,14-20:3 (**Table 1**).

**Table 1:** Average (± standard error) of quantitatively prominent fatty acids of total lipids (% of total FAs) in phytoperiphyton, n = 38, *Apatania crymophila*, n = 21, *Eulimnogammarus viridis*, n = 39, Siberian grayling *Thymallus arcticus*, n = 16 and water moss *Fontinalis antipyretica*, n = 4, from the littoral of the Yenisei River near Krasnoyarsk, 2008–2011.

Fatty acid	periphyton			trichopterans			gammarids			grayling			moss		
14:0	5.5	_±_	0.34	2.5	_±_	0.13	3.1	_±_	0.15	2.0	_±_	0.19	1.1	_±_	0.25
i15:0	0.4	_±_	0.03	0.2	_±_	0.02	0.4	_±_	0.03	0.2	_±_	0.02	n.d.		
15:0	0.4	_±_	0.02	0.2	_±_	0.02	0.3	_±_	0.01	0.2	_±_	0.02	0.4	_±_	0.07
16:0	17.2	_±_	0.60	18.9	_±_	0.71	17.0	_±_	0.53	16.8	_±_	1.24	10.7	_±_	2.12
16:1n-9	0.5	_±_	0.08	0.3	_±_	0.04	0.2	_±_	0.02	0.2	_±_	0.02	n.d.		
16:1n-7	18.0	_±_	1.19	18.1	_±_	0.91	13.7	_±_	0.39	6.0	_±_	0.52	1.1	_±_	0.23
16:1n-5	0.5	_±_	0.05	0.3	_±_	0.05	0.4	_±_	0.04	0.3	_±_	0.04	n.d.		
16:2n-4	2.7	_±_	0.15	2.7	_±_	0.19	1.8	_±_	0.07	0.6	_±_	0.08	0.2	_±_	0.07
16:3n-4	3.0	_±_	0.24	1.7	_±_	0.16	1.6	_±_	0.08	0.5	_±_	0.07	n.d.		
16:3n-3	1.4	_±_	0.16	1.5	_±_	0.17	0.4	_±_	0.03	0.2	_±_	0.03	4.1	_±_	0.81
16:4n-3	2.5	_±_	0.44	5.5	_±_	0.33	0.4	_±_	0.05	0.1	_±_	0.01	0.4	_±_	0.07
16:4n-1	3.6	_±_	0.34	1.7	_±_	0.18	1.5	_±_	0.10	0.2	_±_	0.04	n.d.		
18:0	1.2	_±_	0.10	3.3	_±_	0.27	1.9	_±_	0.09	3.5	_±_	0.27	0.8	_±_	0.25
18:1n-9	3.0	_±_	0.30	9.9	_±_	0.39	16.6	_±_	0.61	12.4	_±_	1.02	1.1	_±_	0.19
18:1n-7	2.7	_±_	0.21	2.0	_±_	0.09	4.3	_±_	0.13	3.2	_±_	0.26	1.1	_±_	0.36
18:2n-6	3.7	_±_	0.38	3.1	_±_	0.37	2.4	_±_	0.08	2.0	_±_	0.18	8.6	_±_	1.15
18:3n-6	0.6	_±_	0.06	0.4	_±_	0.03	0.5	_±_	0.04	0.1	_±_	0.02	3.7	_±_	0.73
18:3n-3	5.2	_±_	0.49	7.6	_±_	0.67	2.4	_±_	0.13	2.0	_±_	0.19	13.2	_±_	2.38
18:4n-3	4.2	_±_	0.65	2.1	_±_	0.23	1.5	_±_	0.12	0.8	_±_	0.09	0.8	_±_	0.12
20:1n-9	0.4	_±_	0.06	0.1	_±_	0.05	0.5	_±_	0.04	0.4	_±_	0.07	0.1	_±_	0.03
6a,9,12-18:3	0.1	_±_	0.03	0.1	_±_	0.04	0.2	_±_	0.07	n.d.			22.3	_±_	1.23
20:4n-6	0.8	_±_	0.07	0.6	_±_	0.07	1.4	_±_	0.07	2.1	_±_	0.17	2.3	_±_	0.36
20:4n-3	0.3	_±_	0.02	0.2	_±_	0.02	0.3	_±_	0.01	0.6	_±_	0.04	n.d.		
20:5n-3	14.4	_±_	0.88	10.0	_±_	0.57	16.0	_±_	0.42	9.3	_±_	0.71	2.7	_±_	0.58
8a,11,14-20:3	n.d.			n.d.			0.2	_±_	0.07	n.d.			5.1	_±_	1.07
22:5n-3	0.7	_±_	0.07	0.1	_±_	0.03	1.2	_±_	0.07	3.3	_±_	0.25	0.4	_±_	0.13
22:6n-3	0.9	_±_	0.08	0.2	_±_	0.04	2.1	_±_	0.15	20.2	_±_	1.67	n.d.		

n.d. – not detected.

**Figure 1:**
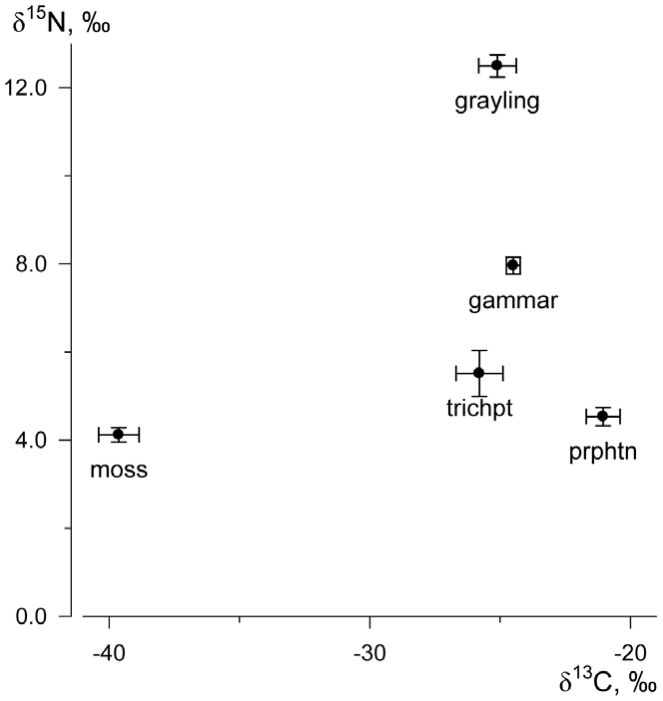
Average values of the isotope ratios (‰) in phytoperiphyton (prpht), *Apatania crymophila* (trchpt), *Eulimnogammarus viridis* (gammar), Siberian grayling *Thymallus arcticus* and water moss *Fontinalis antipyretica* from the littoral of the Yenisei River near Krasnoyarsk, 2008–2011.

The isotope ratio of bulk carbon of the phytoperiphyton (epilithic microalgae) had the highest value (**Figure 1**). δ^13^C values of the zoobenthos, trichopterans and gammarids, and the grayling (**Figure 1**) were nearly identical and significantly lower, than that of phytoperiphyton according to Student's *t*-test (all data sets had normal distribution according to Kolmogorov-Smirnov *d*-test). The water moss, *Fontinalis*, had extremely light carbon isotope composition (**Figure 1**).

On the basis of bulk nitrogen isotope composition (**Figure 1**) trophic positions of consumers were calculated using Equation 1. Animals with the lowest δ^15^N, the trichopteran larvae, were taken as the baseline and thereby got TP = 2 (consumer I). For the gammarids trophic position value was 2.7, and for grayling TP = 4.1. Thus, the gammarids appeared to have nearly third trophic level (consumer II) and the grayling occupied exactly forth trophic level (consumer III) in the ecosystem.

**Figure 2:**
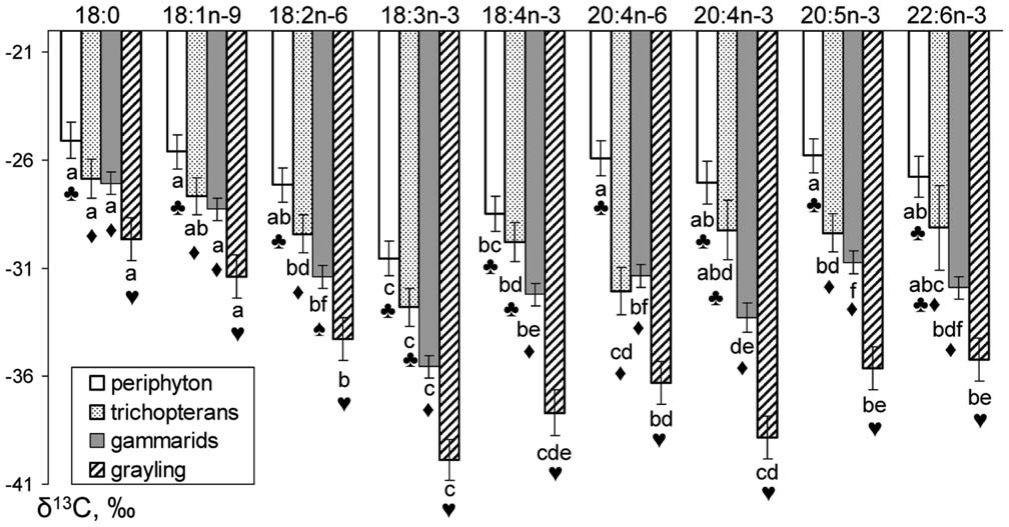
Average isotope composition of fatty acids in phytoperiphyton, trichopterans *Apatania crymophila*, gammarids *Eulimnogammarus viridis* and Siberian grayling *Thymallus arcticus* from the littoral of the Yenisei River near Krasnoyarsk, 2008–2011. Bars represent standard errors. Bars labeled with the same letter are not significantly different within species, and bars labeled with the same symbol are not significantly different between species at P<0.05 after Fisher's LSD *post-hoc* test.

Isotope signatures of nine fatty acids of total lipids in the phytoperiphyton, the zoobenthos and the fish are given in **Figure 2**. In all organisms the essential 18:3n-3 was significantly more depleted, than the other C_18_ acids, except 18:4n-3 in the phytoperiphyton and grayling (Figure 2). 18:3n-3 was also significantly more depleted than C20-C22 acids, except 20:4n-6 and 22:6n-3 in the trichopterans, and 20:4n-3 in the grayling (**Figure 2**). The other essential fatty acid, 18:2n-6, had significantly lower δ^13^C values than 18:0 and 18:1n-9 in gammarids and grayling (**Figure 2**). Apart from 18:3n-3, the other C18 fatty acids in all organisms had nearly the same δ^13^C values as those of C20-C22 acids (**Figure 2**). The contents of ^13^C in 20:4n-6 in all organisms didn't differ significantly from 20:5n-3 and 22:6n-3 (**Figure 2**). 20:5n-3 in the gammarids and the grayling was significantly more enriched, than 20:4n-3. Interestingly, 18:2n-6 didn't differ from 20:4n-6 (**Figure 2**). 20:5n-3 and 22:6n-3 within all organisms had about equal δ^13^C values (**Figure 2**).

Between organisms (trophic levels) differences in δ^13^C values of individual fatty acids in general consisted in depletion of FAs from the first (phytoperiphyton, primary producers) to the forth (grayling, consumers III) trophic level (**Figure 2**). Average δ^13^C values of all acids of the trichopterans (consumers I) were lower, than those of relevant acids of the primary producers (phytoperiphyton), although the differences between 18:3n-3, 18:4n-3, 20:4n-3 and 22:6n-3 were statistically insignificant (**Figure 2**). Average δ^13^C values of the gammarids (consumers II) FAs were lower, than those of the trichopterans, except 20:4n-6, but the differences between 18:0, 18:1n-9, 20:5n-3 and 22:6n-3 were statistically insignificant. All the fatty acids of the grayling (consumers III) were significantly more depleted than those of the gammarids (**Figure 2**).

**Figure 3:**
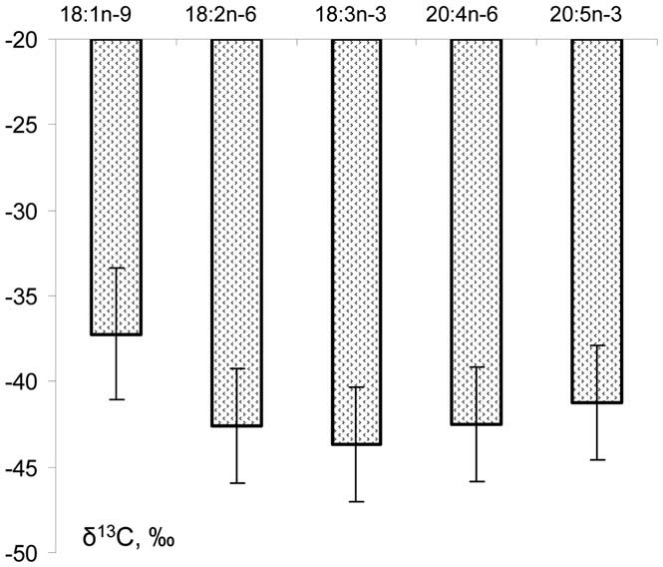
Average isotope composition of fatty acids in water moss *Fontinalis antipyretica* from the littoral of the Yenisei River. Bars represent standard errors.

The data on *Fontinalis* were placed in separate graph (**Figure 3**), because there were less fatty acids in CSIA, and these acids had significantly lower isotope ratios. Thus, if to place too depleted moss acids in one axe with those of the other organisms, important differences between the latter's would not be conspicuous. Average δ^13^C value of 18:3n-3 in the moss was lower, than those of the other acids (**Figure 3**). However, all the differences between average isotope ratios of the fatty acids were statistically insignificant, likely because of the small number of samples. In contrast, δ^13^C average value of each fatty acid in the moss (**Figure 3**) was significantly lower according to Fisher's *post-hoc* test than that of relevant acid of all other organisms (**Figure 2**), i.e. the periphytic microalgae, the trichopterans, the gammarids and the grayling.

In addition, we compared isotope signatures of FA of total lipids with those of triacylglycerols (TAG) in several samples of the gammarids through the studied period. There were no significant differences between δ^13^C values of TAG and total lipids for each fatty acid according to both parametric and non-parametric tests (**Table 2**).

**Table 2:** Average (± standard error) δ^13^C values (‰) of fatty acids (FA) of total lipids and triacylglycerols of *Eulimnogammarus lacustris* from littoral sites of the Yenisei River and significance of their differences according to Student's *t*-test for dependent pairs and Wilcoxon matched pairs T-test for number of pairs n = 9.

FA	Total lipids			Triacylglycerols			*t*	T
18:0	−26.6	±	1.0	−26.7	±	0.7	0.084	18.0
18:1n-9	−28.6	±	1.5	−27.9	±	0.8	0.474	15.0
18:2n-6	−32.0	±	1.9	−32.4	±	1.1	0.221	21.0
18:3n-3	−36.8	±	1.1	−35.8	±	0.9	0.990	15.0
18:4n-3	−32.5	±	0.9	−33.7	±	0.8	2.057	6.0
20:4n-6	−29.9	±	1.2	−29.9	±	1.0	0.052	22.0
20:4n-3	−35.3	±	3.8	−28.1	±	4.0	0.466	17.0
20:5n-3	−29.8	±	1.1	−29.3	±	0.7	0.532	20.0
22:6n-3	−27.8	±	1.1	−28.6	±	1.1	0.172	17.0

There were no significant correlations between δ^13^C values of individual fatty acids the phytoperiphyton vs. the gammarids and the trichopterans.

## Discussion

Fatty acid composition of studied organisms and groups did not differ from those obtained in previous periods [14], [18], [22]. According to bulk nitrogen isotope composition, the trichopteran larvae *A. crymophila* occupied the second trophic level in the ecosystem, i.e. they were consumers I and grazed the phytoperiphyton (epilithic microalgae). Indeed, using fatty acid composition of storage lipids, triacylglycerols (TAG), it was found that the trichopterans in the Yenisei River primarily consumed diatoms and green algae [18], which were dominant species of the phytoperiphyton in the studied sites of the river [14]–[17]. Besides the microalgae, the trichopterans in the studied sites were found to consume a small amount of the water moss, *F. antipyretica*, which had absolutely specific biomarkers, acetylenic fatty acids [22]. However, the levels of the acetylenic fatty acids in trichopterans were about 1%, while levels of sum of these acids in *F. antipyretica* was on average 26% [22]. For comparison, levels of the FA markers of diatoms in trichopterans and in epilithic biofilms at the studied site had practically the same value, around 30–50% [22]. Thereby, it was concluded, that the water moss was the minor part of the trichopterans' diet [22]. Larvae of species of *Apatania* genus are known to be scrapers, which prefer periphytic microalgae [28], just like in our study.

Trophic position of the gammarids, *E. viridis*, was 2.7, i.e. a little less than the third trophic level. *Eulimnogammarus* (*Philolimnogammarus*) species are generalist omnivores [29]. In our study *E. viridis* also was found to be omnivorous species with a high degree of predation, like many other gammarids [30]. Probably, the gammarids obtained a part of carbon directly from the epilithic microalgae [18]. Cannibalism also can be characteristic of many gammarid species [30]. As found previously on the basis of low level, about 1%, of the acetylenic fatty acids in the gammarids, a minor part of ration of the gammarids consisted of the water moss, *F. antipyretica* [22]. This moss-derived carbon, which had extremely depleted isotope content, probably caused the lower bulk δ^13^C values of the gammarids, as well as the trichopterans, compared to that of the phytoperiphyton, found in our study.

The grayling occupied nearly exactly the forth trophic level (TP = 4.1). The fish had practically the same bulk δ^13^C values as the zoobenthos organisms. According to visual gut content analysis an FA marker analysis, the zoobenthos, especially the gammarids, were known to be the principal food items of this fish in the studied site [19]. As known, trophic enrichment, Δδ^15^N, can vary strongly from the common average value of 3.4‰ [24], [25]. However, in our study assuming the conventional value of the constant Δδ^15^N = 3.4‰, we did accurately specify trophic positions of the studied animals, which were in complete agreement with the literature data on their feeding, cited above.

Thus, in general the studied ecosystem encompassed the four-link trophic chain, based on the periphytic microalgae. However, there was a small contribution of the water moss, and the gammarids were omnivorous, although had a high degree of predation, i.e. they roughly had the third trophic level. Fatty acids were transferred through this trophic chain, where the contribution of water moss was negligible [22].

There was an explicit general pattern of FA isotope composition through the trophic levels. Firstly, a gradual depletion of δ^13^C values of fatty acids, including essential, occurred upward trophic levels, from the primary producers (periphytic microalgae) to the consumers III (grayling). Secondly, a dependence of δ^13^C values of fatty acids on degree of unsaturation/chain length in each group of organisms (trophic level) was parabolic, with essential acids 18:2n-6 and 18:3n-3 in its lowest point (**Figure 2**). The same parabolic dependence was also tended to be characteristic of the water moss, although it was statistically insignificant, likely because of the small number of samples (**Figure 3**). Thus, the pattern in the δ^13^C differences between individual fatty acids in the studied river community was absolutely the same, as the pattern, described by Veefkind [4] for marine pelagic food chains.

Besides the data of Veefkind [4], Parrish et al. [31] reported the depletion of HUFA in three-link laboratory food chain protist → rotifer→ cod. Many authors also found a depletion of fatty acids of consumers in ^13^C compared to those of their food. For instance, in laboratory experiments two species of planktonic foraminifera were depleted in ^13^C relative to the corresponding fatty acids from their food, nauplii [32]. Shrimps from Hong Kong streams, *Caridina cantonensis*, fed on phytoperiphyton, had significantly more depleted essential α-linolenic acid (18:3n-3, ALA), than that of the periphyton [33]. Rhee et al. [34] when studied a controlled humans diet, found that the ^13^C content of essential linoleic acid (18:2n-6) in serum was about 2‰ lower, than that of the diet, in contrast to non-essential saturated and monounsaturated acids. Bec et al. [6] found that in laboratory experiment FA in *Daphnia* lipids were generally ^13^C-depleted compared with their counterpart in the corresponding diet, including essential HUFA. Thus, a depletion of essential fatty acids of consumers in ^13^C content compared to those of their food sources, occurred in the studied trophic chain in the Yenisei River, appeared to be a widespread common phenomenon.

Hence, our present findings and many literature data contradict the premise of CSIA that isotope ratios of essential fatty acids in consumers reflect those of their food source, used in some studies [2]. Many authors explained such differences of EFAs between consumers and their studied food sources by an ‘additional unknown unique food source’ [9], [12]. However, Veefkind [4], generalizing original and literature data on marine ecosystems, pointed out, that it would be surprising if organisms of different trophic levels from different environments all had had a similar “unique input” to produce the comparable δ^13^C pattern. Our data on the same pattern in the δ^13^C differences between individual fatty acids in the community of the Yenisei River strongly support the above conclusion of Veefkind [4]. Moreover, in the cases of the controlled diets [6], [31], [34] there were no unknown food sources.

In our study the additional minor food source, water moss, might be responsible for the significant depletion of EFA in the trichopterans and the gammarids compared to those of their main food, periphytic microalgae. However, on the basis of our previous data [22], cited above, we think that it was not the case, and the water moss contribution really was negligible. Moreover, the grayling in the studied site did not graze on the moss and had not an unknown food source. The gammarids were the major food sources for the grayling, because they had about ten-fold higher biomass, than all the other zoobenthos species [14], [19]. The grayling had significantly lower δ^13^C values of fatty acids than its major food source, the gammarids, like consumers in many laboratory experiments and field studies, cited above. Hence, basing on our data on the trophic chain ‘periphytic microalgae → trichopterans → gammarids → grayling’ and on numerous literature data cited above, we can consider depletion of isotope signatures of FA in consumers versus their diet as a common phenomenon. We do not know now how variable is such depletion in diverse trophic chains. More work is evidently needed to conceive patterns and variability of trophic depletion in FA isotope signatures among different consumers in different ecosystems. In any case, the probable trophic depletion should be taken in consideration and at least roughly estimated before one tends to use CSIA for tracing a particular trophic chain.

The depletion of essential fatty acids in consumers evidently cannot be explained by the kinetic isotope effect, since EFAs are not synthesized by almost all consumers. Thus, differences between EFA δ^13^C values in diet and in consumer tissue is believed to be caused by isotopic fractionation occurring during assimilation, transport, or catabolism, i.e., by digestive physiology of the animal [6], [35]. Rhee et al. [34] also speculated that ^12^C linoleic acid is discriminated against at the first committed step in β-oxidation. Thus, animals likely tended to anabolize only ‘high-quality’ light EFA, and catabolize ‘low-quality’ heavy species. As a result, they have to insert lighter EFA in lipid moiety of their tissues. Indeed, Bec et al. [6] hypothesized, that the isotope differences between the diets' and the consumers' lipids are expected for FA that might be synthesized *de novo* or result from the elongation or desaturation of FA precursors, as elongase and desaturase enzymes preferentially use the lighter precursor. Above hypotheses on digestive and biochemical fractionation evidently need following experimental verification.

It should be noted, that some researchers used in CSIA fatty acids from a certain lipid fraction, e.g., from polar lipids or neutral lipids [3], [5]. In our work we used FA from total lipids of all studied organisms. However, we consider that the use of total lipids did not affect the general findings. Indeed, when we compared δ^13^C values of fatty acids in total lipids and in TAG of the gammarids, we found no significant differences.

Generalizing the δ^13^C values of fatty acids within all studied organisms, one can see the following tendency (**Figure 2** **and** **3**): 18:0>18:1n-9>18:2n-6>18:3n-3<18:4n-3<20:4n-6<20:5n-3≈22:6n-3 (we discarded 20:4n-3 because it had very low level and thereby comparatively high analytical error). Veefkind [4] gave similar graphs of δ^13^C values of FA vs. chain length/unsaturation degree for marine pelagic communities. The above parabolic tendency with essential acids 18:2n-6 and 18:3n-3 in its lowest point (**Figure 2** **and** **3**) can be found in many literature data. For instance, in humans serum 18:2n-6 was significantly more depleted in ^13^C content than 18:0 and 18:1n-9, as well as 20:4n-6 [34]. C20 and C22 polyunsaturated fatty acids of cultured dinoflagellates *Amphidinium sp.* and *Gymnodinium simplex* were enriched in ^13^C by up to 8‰ relative to C18 fatty acids [36]. Unsaturated C18 acids were lighter, than 18:0 and 20:5 in some zooplankton taxa, *Bosmina*, *Euchlanis* and *Brachionus* [37]. In sinking organic particles in the Mediterranean Sea 18:3n-3 and 18:4n-3, showed more depleted δ^13^C values compare to other fatty acids, including 20:5n-3, 22:6n-3 and especially to 18:1n-9 [38]. 18:2n-6 in wild and cultured European sea bass filets was more depleted compared to 18:1n-9, 20:5n-3 and 22:6n-3 [39]. In gilthead sea bream (*Sparus aurata*) 18:2n-6 was more depleted acid compared to 18:0, 18:1n-9 on the one hand, and 20:5n-3, 22:6n-3 on the other hand [40]. In a cultured diatom algae *Thalassiosira pseudonana* 20:5n-3 and 22:6n-3 were less enriched compared to 18:2n-6 and 18:1n-9 [41]. Budge et al [8] depicted the parabolic pattern for sea birds, eiders, fed on controlled diet.

The first half of this parabolic tendency, namely the significant depletion of the essential 18:2n-6 and especially 18:3n-3 compared to their less saturated precursors, is in a good agreement with the ideas, that the kinetic isotope effect make more unsaturated fatty acids lighter, than their precursors [6], [9], [11]. In contrast, the other half of the found tendency (18:3n-3<18:4n-3<20:5n-3≈22:6n-3) evidently contradicts the above postulate. Interestingly, in plants methyl-end desaturases are responsible for synthesis of 18:2n-6 and 18:3n-3, while the Δ6 desaturase which count from carboxyl end of the molecule, finally takes part in synthesis of 18:4n-3 from 18:3n-3 [42]. Hence, we speculate that the kinetic isotope effect in lipid synthesis might be inherent only for the methyl-end type of desaturases, but not for front-end desaturases. Thereby, 18:4n-3 had heavier isotope composition than 18:3n-3.

**Figure 4:**
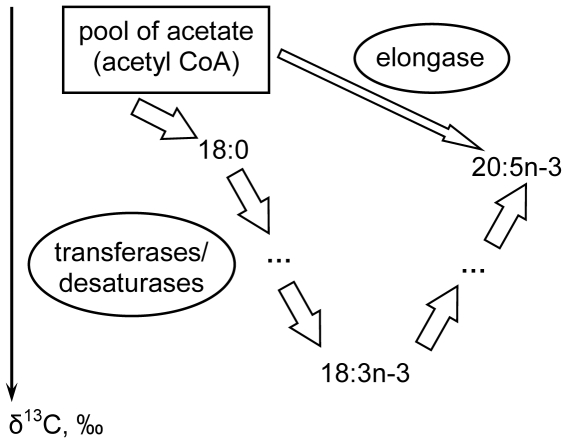
Schematic of synthesis of fatty acids from pool of acetate with relative isotope composition values of the pool and the acids, arranged along δ^13^C axe. Transferases/desaturases provide the kinetic isotope effect, resulting in the isotope depletion in the synthesized fatty acid, 18:3n-3, while elognase adds carbon atom from the acetate pool and thereby provides the isotope enrichment in the synthesized fatty acid, 20:5n-3. A number of intermediate fatty acids are substituted by “…”.

At present it remains unclear why fatty acids such as the 20:5n-3 and 22:6n-3 are so enriched in ^13^C (3–6‰) with respect to the 18:2n-6 and 18:3n-3/4, which are likely their precursors [4]. We can suggest following hypothesis to explain the obtained empirical dependence. Fatty acids are known to be synthesized *de novo* from acetate pool via acetyl CoA, which has a certain δ^13^C value (**Figure 4**). According to the kinetic isotope effect [10], acids with a higher degree of unsaturation (here 18:3n-3), have a lower ^13^C content because of a discrimination of molecules with heavy isotopes by enzymes, e.g., transferases and desaturases during the biosynthesis (**Figure 4**). However, to synthesize polyunsaturated acid with longer chain, here 20:5n-3 from 18:3n-3, elongases must add two carbon atoms, i.e., an acetate unit, to the precursor. Naturally, acetate (acetyl CoA) for the elongation will be taken from the acetate pool. We speculate that the acetate pool is significantly enriched in ^13^C compared to fatty acids. We have following premises for such speculation. Fatty acids are known to be extremely depleted in ^13^C compared to other compounds, i.e., bulk carbon [4], [7], [10]. Indeed, in our study δ^13^C values of bulk carbon of the organisms were ca. 5–15% higher, than those of their fatty acids. Thereby we speculate that the ‘bulk’ acetate pool has a higher δ^13^C than fatty acids (**Figure 4**). Thus, after the elongation by the heavy acetate (acetyl CoA), 20:5n-3 appeared to be heavier in ^13^C content than its precursor, 18:3n-3 (**Figure 4**).

In any case, both the above tendencies, the parabolic empirical dependence together with a possible ‘digestive’ depletion of fatty acids in ^13^C contents upward trophic chains, should be taken into consideration when deciphering CSIA data to disentangling natural trophic food webs. Even in comparatively simple laboratory experiments the interpretation of stable isotopes is complex and it will be difficult to apply this approach to complex field situations without a comprehensive understanding of the factors that determine the δ^13^C values of specific biomarker molecules [26].

Thus, in the zoobenthos community of the Yenisei River we found the pattern in the δ^13^C differences between individual fatty acids, similar to that, described by Veefkind (2003) for marine pelagic food webs. This pattern consists in the gradual depletion of essential FA in consumers compared to their food source, and in the parabolic dependence of δ^13^C values of fatty acids on their degree of unsaturation/chain length. This parabolic dependence was subsequently passed on from primary producers to consumers of all trophic levels.
